# Evaluation of antibiotic susceptibility test results: how guilty a laboratory could be?

**DOI:** 10.1186/s42506-018-0006-1

**Published:** 2019-01-11

**Authors:** Mohamed S. M. Nassar, Walaa A. Hazzah, Wafaa M. K. Bakr

**Affiliations:** Department of Microbiology, High Institute of Public Health, Alexandria University, Alexandria, Egypt

**Keywords:** CLSI, Mueller-Hinton agar, Inhibition zones, Disk diffusion method, Quality control

## Abstract

**Background:**

The selection of an appropriate antimicrobial is a challenging task for clinicians. The Kirby-Bauer disk diffusion method is one of the most widely practiced antimicrobial susceptibility tests (AST). It is affected by many factors among which are the media used. Mueller-Hinton agar (MHA) is the standard medium recommended in guidelines. However, these guidelines are not strictly adhered to in some developing countries.

**Objectives:**

Validation of AST results on nutrient agar (NA) medium used as a substitute for MHA by some microbiology laboratories in Alexandria, Egypt.

**Methods:**

A total of 149 clinical bacterial isolates and 3 reference strains: *Staphylococcus aureus* (*S. aureus*) ATCC® 25923, *Escherichia coli* (*E*. *coli*) ATCC®25922, and *Pseudomonas aeruginosa* (*P. aeruginosa*) ATCC®27853 were comparatively challenged to antibiotics employing MHA and NA.

**Results:**

All antibiotics-reference bacterial strain challenges on NA compared to MHA were unacceptable (> 3 out of limit zones in 30 consecutive days). Considering clinical isolates, the frequency of very major, major, and minor errors on NA was highest in the case of *P. aeruginosa* (8.98%, 4.08%, and 14.7% respectively) followed by *S. aureus* (7.6%, 6%, and 8.8% respectively). On the other hand, the least frequency of errors was in the case of *Enterobacteriaceae* (0%, 0.4%, and 3.2% respectively).

**Conclusions and recommendations:**

Using NA in AST resulted in multiple errors and the high discrepancy in results compared to MHA making it unreliable for susceptibility testing. MHA should not be replaced by NA in AST. Following guidelines and QC measures for AST must be neither bypassed nor underestimated.

## Introduction

Antibiotic resistance has become a serious public health problem all over the world. Nearly two million people in the USA acquire nosocomial infections every year, resulting in 90,000 deaths. More than 70% of the bacteria that causes these infections are resistant to at least one of the antibiotics commonly used in treatment [1]. This makes the selection of an appropriate agent an increasingly more challenging task that has made clinicians more dependent on data from in-vitro AST [2].

In the industrial world, the Kirby-Bauer disk diffusion method is a standard procedure for the susceptibility testing of bacterial isolates. When the test is performed following a standard procedure, it gives reliable results and can predict clinical efficacy of the antibiotics tested [3, 4]. It has been recognized for years that the general adoption of antimicrobial susceptibility test (AST) method, so standardized as to minimize the influence of variables, would be a great advance. The validity of AST depends on rigid standardization of every feature of the test, including particularly the composition of the medium used [5]. The standard medium for the Kirby-Bauer method of susceptibility testing is Mueller-Hinton agar (MHA) [6]. Because of the number of difficulties and financial issues, MHA is not a feasible option in many developing countries, and instead, NA is used for AST [7]. We performed a pilot study on 30 randomly selected clinical microbiology laboratories in Alexandria, using a questionnaire sheet to assess their compliance to Clinical and Laboratory Standards Institute (CLSI) guidelines for AST by the disk diffusion method. Most of the laboratories did not perform internal quality control (QC), and non-adherence to the type of medium was one of the frequent errors encountered. This study aimed at validating the AST results when performed using NA as a substitute for MHA by some laboratories.

## Material and methods

This comparative cross-section study was carried out during a 9-month period from June 2015 through February 2016 at the Microbiology Laboratory of the High Institute of Public Health (HIPH), Alexandria, Egypt.

Three reference bacterial strains, namely *Staphylococcus aureus* (*S. aureus*) ATCC® 25923, *Escherichia coli* (*E. coli*) ATCC® 25922, and *Pseudomonas aeruginosa* (*P. aeruginosa*) ATCC® 27853, were used for the internal QC of AST for 30 consecutive days according to the CLSI guidelines [6]. We performed the internal QC of AST using Oxoid antibiotic disks with reference strains on two media (MHA and NA), followed by comparing the performance of both media in AST of 149 clinical bacterial isolates (Table 1). All clinical bacterial isolates were identified by morphological and biochemical tests according to standard methods [8].

**Table 1:** Clinical bacterial isolates

Clinical bacterial isolates	Number
*Staphylococcus aureus*	50
*Pseudomonas aeruginosa*	49
*Escherichia coli*	17
*Klebsiella pneumoniae*	20
*Proteus vulgaris*	3
*Proteus mirabilis*	10

Zone diameters of susceptibility testing results were categorized as sensitive, intermediate, or resistant based on the CLSI breakpoint criteria [6]. Susceptibility testing results on MHA were compared with those of NA.

Agreements and discrepancies in the results of direct disk diffusion using NA as a medium and MHA were classified as follows: agreement (identical result on NA and MH agar), very major error (susceptible on NA but resistant on MH agar), major error (resistant on NA but susceptible on MH agar), and minor error (susceptible or resistant on NA and intermediate on MH agar, or vice versa) [9, 10].

### Statistical analysis

Data were analyzed using SPSS version 21 (IBM Corp, USA).

## Results

In our study, 30 laboratories were assessed for their compliance with CLSI guidelines for AST by the disk diffusion method. Only three laboratories (10%) were adequately complying with the guidelines. Most laboratories do not perform internal QC or use QC strains, and antibiotic disk brands were used indifferently according to availability and without prior testing. The type of medium used is one of the frequent events encountered (56.7%) causing lack of compliance with the CLSI guidelines for AST. Instead of MHA, NA and tryptic soy agar were used by 11 (36.7%) and 6 (20%) laboratories respectively (results not shown).

Comparing NA to MHA, internal QC testing of the three reference bacterial strains over 30 consecutive days showed that all antibiotics/organism challenges on NA were unacceptable (Table 2). That was reflected on the AST results of clinical isolates cultured on both media (Table 3), where the frequency of very major errors, major errors, and minor errors of NA compared to MH was highest in the case of *P. aeruginosa* (8.98%, 4.08%, and 14.7% respectively) followed by *S. aureus* (7.6%, 6%, and 8.8% respectively). On the other hand, the least frequency of errors was in the case of *Enterobacteriaceae* (0%, 0.4%, and 3.2% respectively).

**Table 2:** Antibiotic susceptibility test results of reference bacterial strains on MH agar versus NA during 30 days of QC testing, Alexandria, 2015–2016

Reference strains	Antibiotic tested	Out of range results/30 QC days* (compared to CLSI ranges)			
		MH		NA	
		Larger IZs	Smaller IZs	Larger IZs	Smaller IZs
*P. aeruginosa* ATCC® 27853	Ceftazidim (CAZ)	0/30	0/30	13/30	2/30
	Gentamicin (CN)	0/30	0/30	4/30	13/30
	Tobramycin (TOB)	0/30	0/30	0/30	15/30
	Piperacillin (PRL)	1/30	2/30	6/30	9/30
	Ciprofloxacin (CIP)	2/30	0/30	19/30	2/30
*E. coli* ATCC®25922	Ampicillin (AMP)	0/30	3/30	0/30	13/30
	Cefazolin (KZ)	0/30	1/30	0/30	13/30
	Gentamicin (CN)	0/30	2/30	0/30	6/30
	Tobramycin (TOB)	0/30	1/30	0/30	9/30
	Tazobactam-piperacillin (TZP)	0/30	0/30	2/30	6/30
*S. aureus* ATCC®25923	Azithromycin (AZM)	0/30	2/30	0/30	23/30
	Clindamycin (DA)	0/30	2/30	0/30	9/30
	Cefoxitine (FOX)	0/30	2/30	6/30	1/30
	Penicillin (P)	1/30	2/30	16/30	2/30
	Sulfamethoxazol-trimethoprim (SXT)	0/30	0/30	4/30	13/30

*QC performed over 30 consecutive days

**Table 3:** Antibiotic susceptibility test results of clinical bacterial isolates on MHA versus NA using CLSI zone size interpretative table HIPH, Alexandria, 2015–16

Antibiotic/clinical isolate	SIR agreement*	Very major error	Major error	Minor error
	No. (%)	No. (%)	No. (%)	No. (%)
*P. aeruginosa* (*n* = 49)				
Ceftazidim (CAZ)	46 (93.90)	0 (0.00)	3 (6.10)	0 (0.00)
Gentamicin (CN)	38 (77.55)	8 (16.30)	2 (4.10)	1 (2.00)
Tobramycin (TOB)	33 (67.30)	10 (20.40)	2 (4.10)	4 (8.20)
Piperacillin (PRL)	24 (49.00)	0 (0.00)	1 (2.00)	24 (49.00)
Ciprofloxacin (CIP)	36 (73.50)	4 (8.20)	2 (4.10)	7 (14.30)
Total	177 (72.24)	22 (8.98)	10 (4.08)	36 (14.70)
*Enterobacteriaceae* (*n* = 50)				
Ampicillin (AMP)	50 (100.00)	0 (0.00)	0 (0.00)	0 (0.00)
Cefazolin (KZ)	46 (92.00)	0 (0.00)	1 (2.00)	3 (6.00)
Gentamicin (CN)	50 (100.00)	0 (0.00)	0 (0.00)	0 (0.00)
Tobramycin (TOB)	47 (94.00)	0 (0.00)	0 (0.00)	3 (6.00)
Tazobactam-piperacillin (TZP)	48 (96.00)	0 (0.00)	0 (0.00)	2 (4.00)
Total	241 (96.40)	0 (0.00)	1 (0.40)	8 (3.20)
*S. aureus* (*n* = 50)				
Azithromycin (AZM)	43 (86.00)	0 (0.00)	3 (6.00)	4 (8.00)
Clindamycin (DA)	41 (82.00)	1 (2.00)	0 (0.00)	8 (16.00)
Cefoxitine (FOX)	33 (66.00)	10 (20.00)	7 (14.00)	0 (0.00)
Penicillin (P)	44 (88.00)	2 (4.00)	4 (8.00)	0 (0.00)
Sulfamethoxazol-trimethoprim (SXT)	33 (66.00)	6 (12.00)	1 (2.00)	10 (20.00)
Total	194 (77.60)	19 (7.60)	15 (6.00)	22 (8.80)

*Category agreement with respect to susceptible (S), intermediate (I), and resistant (R) test results

## Discussion

To evaluate the validity of AST results using NA, QC strains were used for AST in uniform conditions including inoculum density, timing of disk application, temperature of incubation, incubation time, size of the plate, depth of the agar medium, proper spacing of the antibiotic disks, and potency of antibiotic disks. Appropriate QC organisms should be tested daily for all antimicrobial agents routinely included in the antimicrobial battery until a laboratory achieves “satisfactory performance.” CLSI makes the definition of “satisfactory performance” as obtaining unacceptable results in no more than 1 out of 20 or 3 out of 30 results obtained in consecutive test days for each antimicrobial agent/organism combination. Once this satisfactory performance is obtained, a laboratory can convert from daily QC testing to weekly QC testing. If all QC test results are within the acceptable limits, the laboratory can continue weekly testing; however, on occasions when a modification in the test is made, consecutive QC testing is required [6].

All antimicrobial agent-reference organism combinations using NA were unacceptable compared to MHA. All antibiotic-organism combination showed more than three misreading over 30 successive days; hence, none of the results would be accepted according to CLSI guidelines.

Muller-Hinton agar is a loose agar that allows for better diffusion of the antibiotics than most other media. A better diffusion leads to a truer zone of inhibition [11]. This criterion was missing in NA manifested by smaller IZs with TOB, CN, and P when testing *P. aeruginosa* ATCC® 27853, AZM and DA when testing *S. aureus* ATCC® 25923, and AMP, KZ, TOB, and CN when testing *E. coli* ATCC® 25922 (Table 2). Both the para-aminobenzoic acid (PABA) and thymine/thymidine content in MHA are reduced to a minimum, thus markedly reducing the inactivation of sulfonamides and trimethoprim when the medium is used for testing the susceptibility of bacterial isolates to these antimicrobics [11]. Thirteen errors with smaller inhibition zone over 30 consecutive days were detected on testing SXT for *S. aureus* ATCC® 25923 using NA (Table 2).

The inability of AST to determine a susceptible result for an organism that is susceptible to the antimicrobial agent being tested is considered a major error (false resistant). Conversely, the inability to detect resistance is assessed as “very major error” (false sensitive). Concerning CLSI guidelines, there is a minimum level of acceptable interpretative errors in susceptibility testing which are quite restrictive. Very major errors should not exceed 1.5%, while major errors should not exceed 3.0%, and overall categorical agreement should equal or exceed 90% for each organism antibiotic challenge [10, 12, 13].

In our study, the discrepancies between the susceptibility results obtained by NA and the standard MHA were obvious when testing clinical isolates with total errors of 27.76%, 22.4%, and 3.6% with *P. aeruginosa*, *S. aureus*, and *Enterobacteriaceae* respectively. Also, very major errors and major errors were beyond the acceptable level of CLSI guidelines (8.98% and 7.6% very major errors and 4.08% and 6% major errors for *P. aeruginosa* and *S. aureus* isolates respectively).

Very major error may lead to the initiation of inadequate antimicrobial therapy and may have fatal consequences especially in severely ill patients where these antibiotics are common first-line substances. On the other hand, major errors deprive the patients of treatment with an effective antibiotic and lead to the use of second-choice drugs, usually more recent and expensive, and thus contribute to economic losses and the selection of resistant strains [12].

Although none of the AST results for *E. coli* ATCC® 25922 was acceptable when using NA (Table 2), we found a remarkable unexplained agreement in AST results of *Enterobacteriaceae* on both NA and MHA (96.4%) with no very major error and 0.4% major error (Table 3). Even if the AST results showed full agreement as in the case of *Enterobacteriaceae*, the issue of lacking data on specific breakpoints concerning the use of NA remains.

Because NA is a general purpose medium rather than standard susceptibility testing medium, there is hardly any data comparing it with MHA in susceptibility testing. Donkor et al. compared NA with MHA in antimicrobial susceptibility testing of *Salmonella* Typhi and *S. aureus* isolates. They reported that the overall discrepancy in susceptibility results between NA and MHA was 8.9%, and this discouraged the use of NA in the Kirby-Bauer method as practiced by some laboratories, due to the considerable error margin this medium may introduce into susceptibility results [14].

Though our results cannot be generalized on the laboratories’ performance as it was a pilot study conducted on a small number of microbiology laboratories and focusing on one factor affecting AST, it highlights the great importance of standardizing every feature in this test to improve the validity of AST reporting of a laboratory.

## Conclusion and recommendations

The high deviation of AST results observed between MHA and NA raises doubts about the reliability of the NA for susceptibility testing and should not be used as a substitute for MHA. Lack of adherence to strict standardized AST seriously affects treatment strategies for the infections by misleading the physician about the sensitivity and resistance of the isolate to different antibiotics with a deleterious effect on the patients’ outcome. Hence, strict adherence to the guidelines and QC measures for AST must be neither bypassed nor underestimated.
